# Vascular hyporesponsiveness to norepinephrine is a major but not exclusive determinant of mortality in septic shock

**DOI:** 10.1186/s13613-025-01520-5

**Published:** 2025-07-14

**Authors:** Antoine Goury, Zoubir Djerada, Jean-Louis Teboul, Olfa Hamzaoui

**Affiliations:** 1 Unité de Médecine Intensive et Réanimation Polyvalente, CHU Reims, Reims, F- 51100 France; 2 Université de Reims Champagne-Ardenne, Unité HERVI Hémostase et Remodelage Vasculaire Post-Ischémie - EA 3801, Reims, F-51100 France; 3 Faculté de Médecine Paris-Saclay, Université Paris-Saclay, Le Kremlin- Bicêtre, France

**Keywords:** Septic shock, Norepinephrine, Diastolic arterial pressure, Mortality, Vascular responsiveness, Heart rate

We thank Dr Enrique Monares-Zepeda and colleagues for their comments and interest in our study “Ability of diastolic arterial pressure to better characterize the severity of septic shock when adjusted for heart rate and norepinephrine dose” [1].

In this study, after conducting a post hoc analysis of the ANDROMEDA cohort, which included 424 patients in septic shock [2], we showed an inverted J-shaped relationship between in-hospital mortality and the index of vascular responsiveness to norepinephrine (VNERi), with a nadir point at 6.7. The first cut-point defining the descending segment of the inverted J-shaped curve was a VNERi of 2.6. The second cut-point, defining the ascending segment, was a VNERi of 10.8. Based on these cut-points, we identified three distinct patient subgroups with different outcomes. Indeed, in-hospital mortality was 56 ± 6% in subgroup 1 (*n* = 214), compared to 28 ± 7% in subgroup 2 (*n* = 182) (*p* < 0.0001) and 39 ± 19% in subgroup 3 (*n* = 28) (*p* < 0.0001).

Dr Enrique Monares-Zepeda and colleagues highlighted a surprising result of our study because the subgroup 3 had higher in-hospital mortality compared to subgroup 2 although a lower infusion of norepinephrine dose in this group with 0.05 $\:\mu\:$g/kg/min [interquartile range (IQR);0.04–0.06] vs. 0.14 $\:\mu\:$g/kg/min [IQR; 0.13–0.15] (*p* < 0.001). In their letter, they explain that one possible explanation regarding this mortality rebound would be that the subgroup 3 had experienced a different management of sepsis shock with more resuscitation fluid and lower infusion of norepinephrine. This strategy could have led to a decrease in the (diastolic arterial pressure) DAP- (central venous pressure) CVP gradient and worsening of renal lesions and morbidity [3].

Based on data from our cohort, this interesting hypothesis is not verified, as there is no difference in CVP with 8 mmHg [IQR; 5–12] vs. 8 mmHg [IQR; 5–14] (*p* = 0.897) or fluid balance in the first 8 h with 1110 mL [IQR; 427–2050].

vs. 922 mL [IQR; 162–2088] (*p* = 0.712) between subgroup 2 and 3 respectively. Furthermore, there is no more acute kidney injury in subgroup 2 with 71% vs. 68% in subgroup 3 (*p* = 0.461).

A compelling hypothesis was a higher proportion of patients with septic myocardial involvement or related cardiovascular comorbidities and/or long-term beta-blocker treatment, which would partly explain the significant decrease in heart rate (HR) in subgroup 3 with HR = 84 [IQR;67–94] vs. HR = 96 [IQR;82–110] in subgroup 2 (*p* = 0.02). However, although subgroup 3 had a higher proportion of patients with a history of acute heart failure (18% vs. 8% in subgroup 2) and coronary artery disease (7% vs. 3% in subgroup 2), these differences did not reach statistical significance (*p* = 0.156 and *p* = 0.236, respectively), likely due to limited statistical power related to the small sample size in subgroup 3 (*n* = 28). The main limitation to exploring this hypothesis in detail is the lack of available hemodynamic data in the ANDROMEDA database. This could justify conducting future prospective studies to validate or refute this hypothesis.

Finally, although vasoplegia and vascular hyporesponsiveness to norepinephrine as reflected by our VNERi ratio are important contributors, they cannot fully account for the mortality associated with septic shock, which remains a multifactorial condition [4]. Indeed, when performing a multivariate analysis of mortality risk factors after adjusting for the VNERi ratio, the two variables that were significantly associated with mortality in subgroup 3 were age with an odds ratio (OR) = 3.14 [IC95%; 1.22–8.09] (*p* = 0.017) and the number of mechanical ventilation free days with an OR = 0.003 [IC95%; 0.0007–0.0013] (*p* < 0.0001)).

In the second part of their letter, Monares-Zepeda et al. propose another statistical model associated with mortality defined by DAP / (VO_2_max x Norepinephrine dose) where VO2max equals (220-age)/(HR). We compared this model with the VNERi model in the ANDROMEDA cohort and retested it after 1,000 bootstraps. The overall performance of the model proposed by Monares-Zepeda et al. is less robust with an Akaike Information Criterion (AIC) of 525 compared to an AIC of 509 for VNERi (delta AIC = 16, *p* < 0.001). This model was not as effective as VNERi for other statistical markers such as likelihood-ratio chi square = 76 vs. 89 and discrimination index R^2^ = 0.220 vs. 0.254, also including the calibration with a mean absolute error of 0.027 vs. 0.023 respectively. Their model, like ours, depicted as an inverted J-shaped relationship but does not provide additional discriminatory information. We have shown the representation of these two respective models in Fig. 1.

**Fig. 1:**
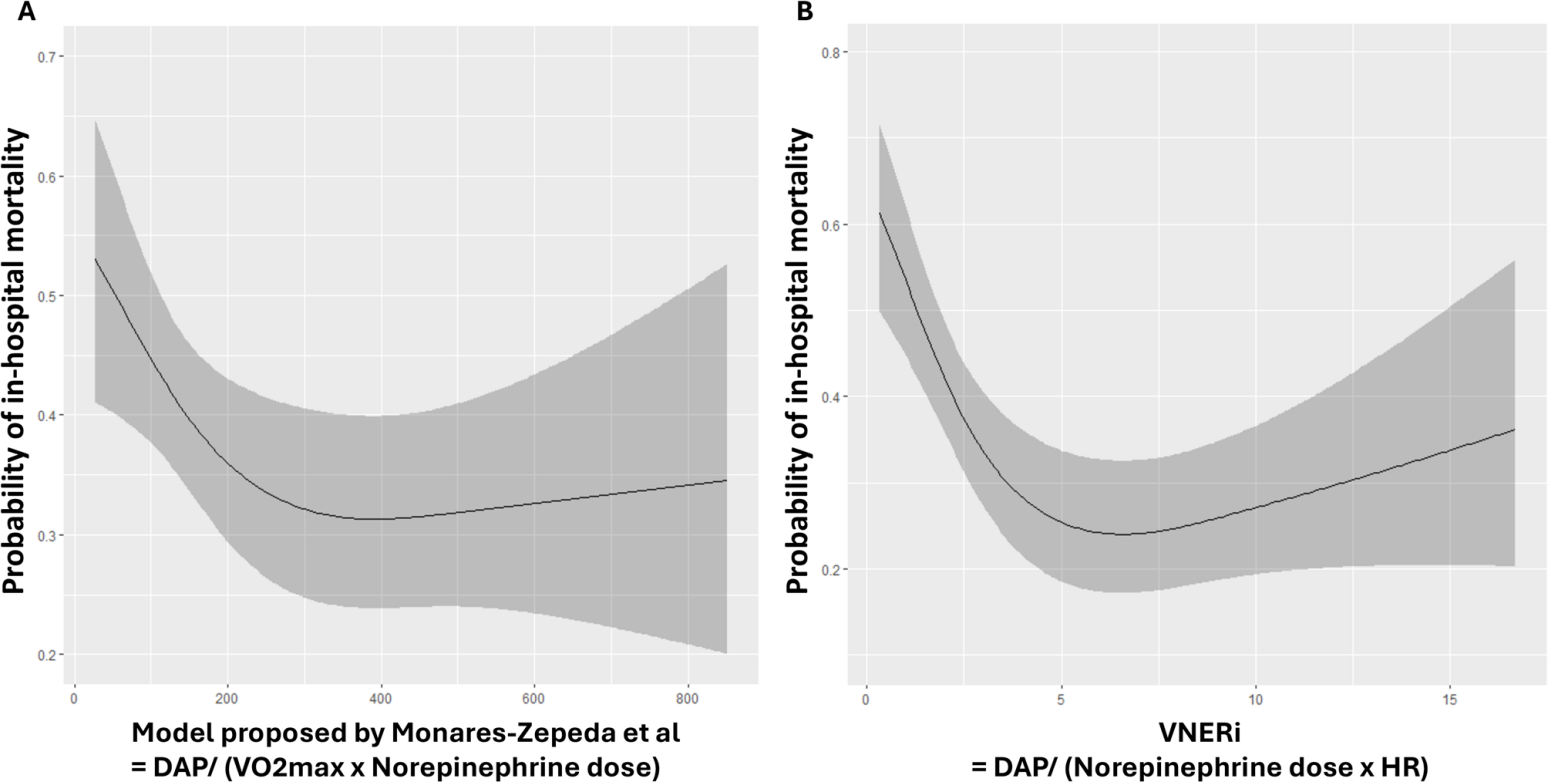
Probability of in-hospital mortality as a function of the Model proposed by Monares-Zepeda et al. (= DAP/ (VO2max x Norepinephrine dose)) and the VNERi Abbreviations: DAP: diastolic arterial pressure, HR: heart rate, VNERi: the index of vascular responsiveness to norepinephrine (= DAP/ (Norepinephrine dose x HR)). The multivariate regression model revealed an inverted J-shaped relationship between in-hospital mortality and **(A)** the Model proposed by Monares-Zepeda et al. and **(B)** VNERi. The overall performance of the model proposed by Monares-Zepeda et al. is less robust with an Akaike Information Criterion (AIC) of 525 compared to an AIC of 509 for VNERi (delta AIC = 16, *p* < 0.001). Multivariate analysis model was adjusted for the baseline covariates: age, sex, weight, APACHE II and SOFA score, pre-randomisation resuscitation fluid volume in the ANDROMEDA-SHOCK study, Norepinephrine dose, HR, systolic arterial pressure, mean arterial pressure, DAP, central venous pressure, mottling score, capillary refill time, plasma lactate level, central venous oxygen saturation, carbon dioxide pressure difference between central venous blood and arterial blood.

Finally, this specific pattern linking VNERi to mortality likely reflects the inherent complexity of septic shock and the heterogeneity of patients admitted in intensive care unit. Characterizing the vascular hyporesponsiveness to vasopressors and distinguishing the hemodynamic phenotypes to which these patients belong may represent a critical step toward the development of more individualized and physiologically tailored strategies for the management of septic shock.

## Data Availability

the datasets used and/or analysed during the current study are available from the corresponding author on reasonable request.

## Abbreviations

AIC: Akaike Information Criterion
CVP: Central venous pressure
DAP: Diastolic arterial pressure
HR: Heart rate
IQR: Interquartile range
OR: Odds ratio
VNERi: Index of vascular responsiveness to norepinephrine
